# Interstate Highway Connections and Traced Gun Transfers Between the 48 Contiguous United States

**DOI:** 10.1001/jamanetworkopen.2024.5662

**Published:** 2024-04-09

**Authors:** Leah Roberts, Mark H. Hoofnagle, Brady Bushover, Ariana N. Gobaud, Christina A. Mehranbod, Carolyn Fish, Christopher N. Morrison

**Affiliations:** 1 Department of Epidemiology, Mailman School of Public Health, Columbia University, New York, New York; 2 Department of Surgery, Washington University in St Louis School of Medicine, St Louis, Missouri; 3 Department of Epidemiology and Preventive Medicine, School of Public Health and Preventive Medicine, Monash University, Melbourne, Victoria, Australia

## Abstract

**Importance:**

Interstate gun flow has critical implications for gun violence prevention, as gun transfers across state lines can undermine local gun control policies.

**Objective:**

To identify possible gun trafficking routes along interstate highways in the US.

**Design, Setting, and Participants:**

This repeated-measures, ecological, cross-sectional study used data from the Bureau of Alcohol, Tobacco, Firearms and Explosives from January 1, 2010, to December 31, 2019, to examine associations between interstate connections via 13 highways that each spanned at least 1000 miles and interstate traced gun transfer counts for the 48 contiguous United States. Analyses were completed in November 2023.

**Exposures:**

Characteristics of the origin states and the transportation connections between the destination state and the origin states.

**Main Outcomes and Measures:**

The main outcome was the total count of guns used in crimes in each destination state per year that were originally purchased in the origin state. Bayesian conditional autoregressive Poisson models were used to examine associations between the count of guns used in crime traced to interstate purchases and interstate highway connections between origin and destination states.

**Results:**

Between 2010 and 2019, 526 801 guns used in crimes in the contiguous 48 states were traced to interstate purchases. Northbound gun transfers along the Interstate 95 corridor were greater than expected to New Jersey (incidence rate ratio [IRR], 2.80; 95% credible interval [CrI], 1.01-7.68) and Maryland (IRR, 3.07; 95% CrI, 1.09-8.61); transfers were similarly greater along Interstate 15 southbound, Interstate 25 southbound, Interstate 35 southbound, Interstate 75 northbound and southbound, Interstate 10 westbound, and Interstate 20 eastbound and westbound.

**Conclusions and Relevance:**

This repeated-measures, ecological, cross-sectional study identified that guns used in crimes traced to interstate purchases moved routinely between states along multiple major transportation routes. Interstate gun transfers are a major contributor to gun crime, injury, and death in the US. National policies and interstate cooperation are needed to address this issue.

## Key Points

**Question:**

Are firearms used in crimes transferred between US states connected via major interstate highways beyond what is expected based on spatial proximity?

**Findings:**

In this cross-sectional study, between 2010 and 2019, 526 801 guns used in crimes in the 48 contiguous United States were traced to interstate purchases. Traced gun transfers were greater than expected along Interstate 15 southbound, Interstate 25 southbound, Interstate 35 southbound, Interstate 75 northbound and southbound, Interstate 95 northbound, Interstate 10 westbound, and Interstate 20 eastbound and westbound.

**Meaning:**

The findings suggest that guns used in crimes are transferred routinely between states connected via major interstate highways.

## Teaser

This cross-sectional study investigates whether firearms used in crimes are transferred between states via major interstate highways at rates greater than expected based on spatial proximity.

## Introduction

Interstate gun flow has critical implications for gun violence and public health in the US. From 2010 to 2019, 275 345 people died and 803 393 were admitted to emergency departments due to interpersonal shooting events.^1^ In 2019 alone, over 30 000 guns traced to in-state and interstate purchases were used in violent crimes such as assault, robbery, and murder.^2^ In addition to direct associations of gun crime with health and safety, exposure to violence can have lasting consequences, including psychological effects and increased risk of cardiovascular disease and premature mortality.^3^ Public and private authorities at the city, county, and state levels invest heavily in preventive interventions to reduce gun crimes and protect the health of their communities. However, because these jurisdictions are connected geographically, interventions implemented locally can be affected by gun policies in other jurisdictions.

Given that guns are easily transportable, lightweight goods and that there is considerable variation in gun laws between states,^4^ theories of economic geography predict that guns will flow illegally from origin states with fewer restrictions to destination states with more restrictions.^5,6,7,8^ Once in these destinations, they can be used in ways that are damaging to population health, including crimes linked to hazardous substances, various forms of assault, and incidents leading to suicides or homicides.^2^ Prior research by some of us demonstrated that inflow of guns from other states undermines local gun supply–reduction strategies, ultimately draining limited resources and contributing to the overall burden of gun crime in the US.^9,10,11^

A related observation is that gun flow is greatest along key interstate transportation routes.^12,13,14^ This phenomenon has been named the Iron Pipeline and refers most commonly to the Interstate 95 (I-95) corridor. Guns from states with fewer restrictions on gun purchases along this corridor, such as Pennsylvania and Georgia, are more frequently traced to crimes in states with stricter gun laws, such as New York and New Jersey, along the same corridor.^15^ This notion has become so common that references to I-95 as an Iron Pipeline can be found in many sources, including congressional bills,^16,17^ news reports,^18^ and presidential statements.^19^ However, I-95 may not be the only Iron Pipeline. For example, patterns of guns moving from southern states to Illinois and western states into California have been observed.^9^ A critical gap for empirical research is identifying other transportation routes that contribute to gun crimes committed interstate. Additionally, it is imperative that such research accounts for spatial autocorrelation, wherein states that are closer together are likely to be more similar than those that are farther apart.^20^

This study aimed to identify possible gun trafficking routes along US interstate highways. The Interstate Highway System is a vital part of the transportation network, with approximately one-quarter of all vehicle miles traveled in the US occurring on interstate highways.^21^ Reduction of gun trafficking within this network may have critical implications for violence prevention and associated health effects. We hypothesized that counts of traced firearm transfers between states connected via major interstate highways would be greater than what is expected based on spatial proximity and that traced gun transfers would be greatest along the I-95 corridor.

## Methods

### Ethics

This cross-sectional study used only publicly available data and was therefore not considered human participants research by the institutional review board of Columbia University. We followed the Strengthening the Reporting of Observational Studies in Epidemiology (STROBE) reporting guideline.

### Study Setting and Design

The setting for this repeated-measures, ecological, cross-sectional study was the 48 contiguous United States from January 1, 2010, to December 31, 2019. Analyses were completed in November 2023. We conducted separate analyses for each state (ie, destination state), wherein the units of analyses were annual observations for each of the other 47 origin states (ie, 47 states ×10 years = 470 state-years).

### Data

The dependent measure was the count of guns used or suspected to be used in crimes in the destination state per year traced to interstate purchases. Interstate gun trace data from January 1, 2010, to December 31, 2019, were obtained from the Bureau of Alcohol, Tobacco, Firearms and Explosives (ATF) National Tracing Center, which provides aggregated state-by-state reports on firearm recoveries.^2,22^ We used these data to create 48 separate datasets, 1 for each destination state of interest. Each dataset contained 470 rows for each origin state-year under investigation. For example, the New Jersey dataset included 10 rows containing the count of guns recovered in New Jersey that originated in Alabama from 2010 to 2019, 10 rows for Arizona, and so on.

**Figure 1.**
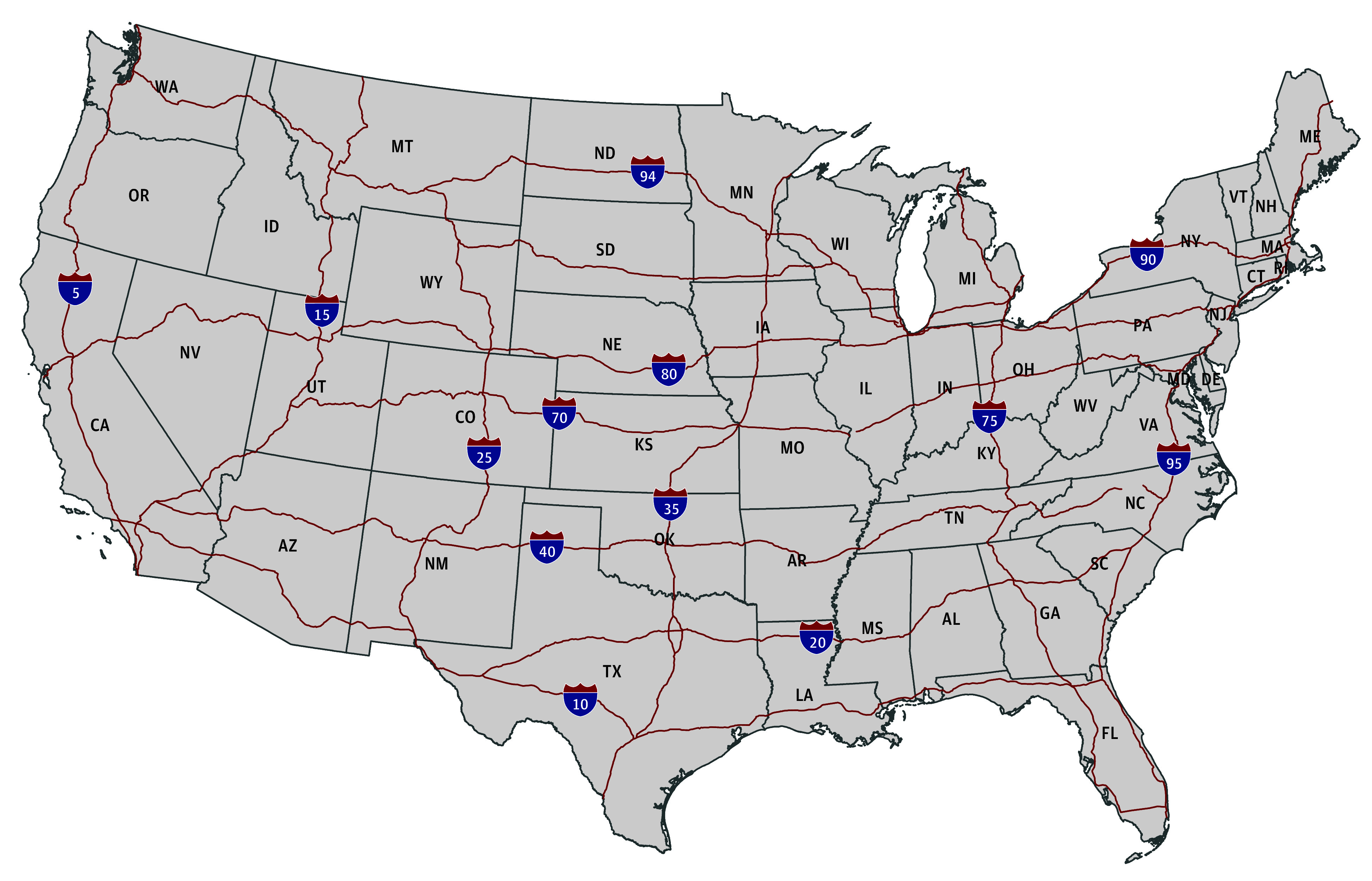
Map of Major Interstate Highways Over the 48 Contiguous United States Lines show the 13 major highways that each span over 1000 miles.

The main independent variable was the directional interstate highway connection. TIGER/Line Shapefiles were used to develop measures of the interstate highway network within the 48 contiguous states.^23^ Thirteen interstate highways that each spanned at least 1000 miles were included: Interstate 90 (I-90), Interstate 80 (I-80), Interstate 40 (I-40), Interstate 10 (I-10), Interstate 70 (I-70), I-95, Interstate 75 (I-75), Interstate 94 (I-94), Interstate 35 (I-35), Interstate 20 (I-20), Interstate 15 (I-15), Interstate 5 (I-5), and Interstate 25 (I-25) (Figure 1). Each of these highways has either northbound-southbound or eastbound-westbound directionality, which was examined separately for a total of 26 highway variables. Highways were coded as 1 if the origin and destination states were connected via that highway in that direction and 0 otherwise. For example, when examining Florida as an origin state and Maryland as a destination state, the I-95 northbound variable would be coded as 1 while the southbound variable would be coded as 0.

We included several independent variables to account for potential confounders: (1) the presence of an interstate connection between the origin and destination states, (2) a corresponding variable for the reverse direction on the same highway (eg, if analyzing I-95 northbound, we included a variable for I-95 southbound), (3) the natural logarithm of the annual population size of each origin state, and (4) the distance between the geometric centroids of the origin and destination states. Coefficients for these variables are not reported. A common observation is that human mobility approximates a geographic gravity function following Newton’s law of universal gravitation, wherein people and goods are more likely to flow from areas with higher populations to areas within a shorter geographic distance.^11,24,25^ While other methods for estimating mobility have been developed,^26^ in the absence of complete data on the movement of people and goods, the gravity function has been established as a strong proxy.^24,25^ Therefore, we expected the count of recovered guns in each destination state to be proportionate to the origin state population size and inversely proportionate to the distance between the origin and destination states.^27^ We refer to this combination of population and distance variables in our model as a *gravity function*. We included the variables for any highway connection between the 2 states to help control for the complexity of travel routes as well as the variable for the opposite direction on the same highway to help isolate the independent association of each directional highway with the count of recovered guns, as we hypothesized that associations may not be the same in both directions.

### Statistical Analysis

All analyses were conducted using R, version 4.3.1 (R Project for Statistical Computing).^28^ We first determined the number of firearms used in crimes in each state that were traced to purchases in other states. Then, we developed models to evaluate the association between the interstate highway network and the number of firearms recovered in each destination state.

We used a Poisson model for observed counts of recovered guns *Y* in state *i* at time *t* using the formula *Y*_i,t_|*u*_i,t_ ~ Poisson(*E*_i,t_exp[*u*_i,t_]), where *E*_i,t_ is the time-varying population size for state *i*. This term serves as the expected number of recovered guns. The mean and variance of the Poisson distribution is *E*_i,t_ multiplied by exp(*u*_i,t_), which is the incidence rate for gun recovery in state *i* at time *t*.

We modeled the log of the incidence rate linearly: *u*_i,t_ = (α + Ω_s_) + β × *X*′_i,t_ + φ_i_ + ω_t_. The term α is an overall intercept, and Ω_s_ is a random intercept capturing time-invariant variation between *s*, the 48 states. Parameter β is a vector of fixed-effect estimates that we interpreted as the associations of interest for a matrix of independent variables *X*′_i,t_, including the directional highway variables, any interstate connection, and the gravity function. The term φ is a conditional autoregressive random effect that controlled for the loss of unit independence, identified using a matrix of adjacent states with queen contiguity. The term ω_t_ captures temporal variation around the average of the linear time trend for all states across the 10 years. We estimated incidence rate ratios (IRRs) from model slopes to report the rate of traced gun transfers to each destination state from states connected vs not connected via the interstate of interest.

We developed individual models for each highway and the corresponding destination states along these routes. Using I-95 northbound as an example, we created specific models for each of the 14 states that are connected by this highway. For instance, in the New Jersey model for I-95 northbound, the primary outcome measured was the number of firearms recovered in New Jersey that originated from the other 47 states. The key independent variable in this case was the connectivity of these origin states to New Jersey via I-95 northbound.

We specified 3 model variants for each state and interstate of interest. Model 1 included only the directional highway variables and the interstate connection variable. Models 2 and 3 were estimated using R-INLA, which calculates the integrated network Laplace approximation of a fully bayesian model.^29,30,31^ Model 2 included the random effect to capture residual variance in traced gun transfers across years and the time-invariant conditional autoregressive random effect to capture spatially structured error. Model 3 included these same spatial and temporal terms as well as the gravity function and was the primary model of interest. The inclusion of both the gravity function and the conditional autoregressive term helped isolate the association of individual interstate highways with traced gun transfers beyond what would be expected based on population size, distance, and adjacency between states.

## Results

### Descriptive Statistics

Between 2010 and 2019, 526 801 guns used in crimes in the 48 states were traced to interstate purchases. Absolute mean (SD) annual traced gun transfers were greatest into California (7012 [2274]), Illinois (4202 [883]), and New York (3545 [366]). Mean (SD) annual traced gun transfers per 1 million population were greatest into Maryland (423 [102]), Illinois (328 [69]), and Delaware (289 [26]). Absolute mean (SD) annual traced gun transfers were greatest out of Georgia (3519 [950]), Texas (3130 [803]), and Florida (2870 [591]). Mean (SD) annual traced gun transfers per 1 million population were greatest out of Mississippi (587 [146]), West Virginia (564 [100]), and Nevada (504 [192]) (Table 1).

**Table 1.** Characteristics of the 48 Contiguous US States, 2010-2019

State	Major interstate connections, No.	Mean (SD) [range]		
		Annual population, millions	Annual traced gun transfers into state, per 1 million population	Annual traced gun transfers out of state, per 1 million population
Alabama	2	4.8 (0.1) [4.7-4.9]	152 (39) [87-203]	389 (95) [297-535]
Arizona	3	6.6 (0.3) [6.2-7.1]	173 (15) [155-198]	370 (99) [261-510]
Arkansas	1	2.9 (0.0) [2.9-3.0]	82 (36) [45-146]	305 (71) [227-411]
California	5	38.1 (0.9) [36.6-39.3]	183 (55) [116-259]	44 (3) [39-49]
Colorado	2	5.2 (0.2) [4.9-5.6]	162 (63) [70-236]	159 (31) [125-208]
Connecticut	1	3.6 (0.0) [3.5-3.6]	82 (25) [59-132]	70 (9) [58-85]
Delaware	1	0.9 (0.0) [0.9-1.0]	289 (26) [250-320]	258 (49) [180-332]
Florida	3	19.6 (0.8) [18.5-20.9]	162 (18) [141-190]	146 (24) [119-177]
Georgia	3	10.0 (0.3) [9.5-10.4]	214 (35) [179-268]	351 (84) [266-486]
Idaho	2	1.6 (0.1) [1.5-1.7]	154 (32) [105-198]	286 (64) [196-406]
Illinois	4	12.8 (0.0) [12.7-12.9]	328 (69) [257-446]	76 (15) [60-103]
Indiana	4	6.5 (0.1) [6.4-6.7]	130 (36) [91-181]	369 (84) [291-527]
Iowa	2	3.1 (0.0) [3.0-3.1]	113 (36) [71-174]	144 (45) [80-210]
Kansas	2	2.9 (0.0) [2.8-2.9]	192 (63) [101-295]	249 (79) [157-366]
Kentucky	1	4.4 (0.1) [4.3-4.4]	164 (51) [108-253]	381 (87) [293-510]
Louisiana	2	4.6 (0.1) [4.4-4.7]	275 (70) [204-397]	272 (61) [207-352]
Maine	1	1.3 (0.0) [1.3-1.3]	50 (8) [38-62]	210 (35) [174-268]
Maryland	2	5.9 (0.1) [5.7-6.0]	423 (102) [288-574]	71 (6) [61-80]
Massachusetts	2	6.7 (0.1) [6.5-6.9]	108 (21) [80-137]	37 (5) [32-48]
Michigan	2	9.9 (0.0) [9.9-10.0]	112 (11) [90-128]	82 (13) [69-100]
Minnesota	3	5.4 (0.1) [5.2-5.6]	109 (30) [66-157]	71 (14) [57-91]
Mississippi	2	3.0 (0.0) [2.9-3.0]	167 (54) [114-243]	587 (146) [422-828]
Missouri	2	6.0 (0.1) [5.9-6.1]	174 (45) [124-248]	191 (76) [105-312]
Montana	3	1.0 (0.0) [1.0-1.1]	135 (39) [87-189]	350 (90) [232-467]
Nebraska	1	1.9 (0.0) [1.8-1.9]	184 (48) [118-260]	127 (21) [104-172]
Nevada	2	2.8 (0.1) [2.6-3.0]	275 (120) [70-486]	504 (192) [294-802]
New Hampshire	1	1.3 (0.0) [1.3-1.3]	43 (11) [31-62]	265 (57) [216-389]
New Jersey	2	8.9 (0.1) [8.7-9.0]	208 (24) [178-243]	26 (4) [22-30]
New Mexico	3	2.1 (0.0) [2.0-2.1]	167 (69) [78-292]	266 (66) [182-357]
New York	2	19.5 (0.2) [19.2-19.8]	181 (17) [161-210]	29 (2) [27-35]
North Carolina	2	9.8 (0.3) [9.3-10.3]	248 (41) [207-314]	218 (32) [183-263]
North Dakota	1	0.7 (0.0) [0.7-0.8]	164 (75) [69-287]	238 (106) [102-381]
Ohio	4	11.6 (0.0) [11.5-11.7]	132 (20) [111-165]	170 (28) [141-215]
Oklahoma	2	3.8 (0.1) [3.7-3.9]	84 (23) [62-125]	241 (52) [189-325]
Oregon	1	3.9 (0.1) [3.8-4.1]	178 (32) [142-238]	207 (34) [162-253]
Pennsylvania	4	12.7 (0.1) [12.6-12.8]	109 (18) [93-139]	158 (21) [127-185]
Rhode Island	1	1.1 (0.0) [1.1-1.1]	104 (26) [62-139]	54 (13) [41-78]
South Carolina	2	4.8 (0.2) [4.5-5.0]	206 (66) [118-321]	409 (81) [322-556]
South Dakota	1	0.8 (0.0) [0.8-0.9]	104 (74) [30-203]	199 (54) [136-273]
Tennessee	2	6.5 (0.2) [6.2-6.7]	271 (106) [132-427]	235 (61) [172-328]
Texas	4	26.3 (1.3) [24.3-28.3]	92 (25) [63-124]	118 (24) [92-157]
Utah	3	2.9 (0.1) [2.7-3.1]	87 (26) [54-130]	176 (36) [132-230]
Vermont	0	0.6 (0.0) [0.6-0.6]	75 (31) [48-149]	257 (69) [157-370]
Virginia	1	8.2 (0.2) [7.8-8.5]	142 (22) [122-182]	329 (62) [265-419]
Washington	2	7.0 (0.3) [6.6-7.4]	113 (21) [91-144]	141 (20) [114-164]
West Virginia	1	1.8 (0.0) [1.8-1.9]	139 (41) [84-192]	564 (100) [442-725]
Wisconsin	2	5.7 (0.1) [5.6-5.8]	92 (18) [73-123]	132 (33) [99-178]
Wyoming	3	0.6 (0.0) [0.5-0.6]	121 (29) [74-156]	416 (120) [262-589]

### Bayesian Conditional Autoregressive Poisson Models

Table 2 reports the results from the I-95 northbound models as an example. Fully adjusted model 3 results for all other highways and states are given in the eTable in Supplement 2. Results for states with low counts of recovered firearms are not reported due to unstable estimates in the spatial models. The state with the largest IRR for I-95 northbound model 1 was New Jersey. The count of guns recovered in New Jersey from states connected to New Jersey via I-95 northbound was greater than the count of guns recovered in New Jersey from states not connected to New Jersey via I-95 northbound when adjusting for the I-95 southbound connection and any interstate connection (IRR, 10.75; 95% CI, 10.17-11.36). After adding spatial and temporal effects in model 2, this IRR decreased to 5.44 (95% credible interval [CrI], 1.52-19.44), and after adding the gravity function in model 3, the IRR decreased further to 2.80 (95% CrI, 1.01-7.68).

**Table 2.** Results of I-95 Northbound Models

State	Incidence rate ratio (95% CI)	Incidence rate ratio (95% CrI)	
	Model 1^a^	Model 2^b^	Model 3^c^
Maine	4.15 (3.54-4.87)	NA^d^	NA^d^
New Hampshire	0.39 (0.28-0.53)	0.73 (0.05-10.91)	0.06 (0.01-0.49)
Massachusetts	3.63 (3.34-3.95)	1.92 (0.62-5.92)	0.99 (0.39-2.52)
Rhode Island	5.03 (4.35-5.82)	NA^d^	NA^d^
Connecticut	1.10 (0.92-1.31)	1.33 (0.11-15.54)	0.07 (0.01-0.52)
New York	7.01 (6.78-7.23)	3.62 (1.21-10.78)	2.24 (0.89-5.73)
New Jersey	10.75 (10.17-11.36)	5.44 (1.52-19.44)	2.80 (1.01-7.68)
Pennsylvania	4.31 (4.14-4.48)	2.78 (1.04-7.42)	1.86 (0.91-3.83)
Delaware	10.46 (9.47-11.56)	NA^d^	NA^d^
Maryland	5.65 (5.45-5.85)	4.54 (1.31-15.74)	3.07 (1.09-8.61)
Virginia	2.90 (2.76-3.04)	2.41 (0.51-11.42)	1.36 (0.45-4.09)
North Carolina	6.66 (6.45-6.87)	3.03 (0.46-19.96)	1.50 (0.42-5.31)
South Carolina	5.45 (5.13-5.79)	3.19 (0.66-15.31)	1.69 (0.56-5.10)
Georgia	3.35 (3.22-3.48)	0.99 (0.05-18.66)	0.36 (0.06-2.17)

Abbreviations: CrI, credible interval; NA, not applicable.

^a^ Included only the directional highway variables and the interstate connection variable.

^b^ Included the random effect to capture residual variance in traced gun transfers across years and the time-invariant conditional autoregressive random effect to capture spatially structured error.

^c^ Included spatial and temporal terms from models 1 and 2 as well as the gravity function.

^d^ Estimate not reported due to unstable output from spatial models as a result of low firearm recovery counts in the state.

**Figure 2.**
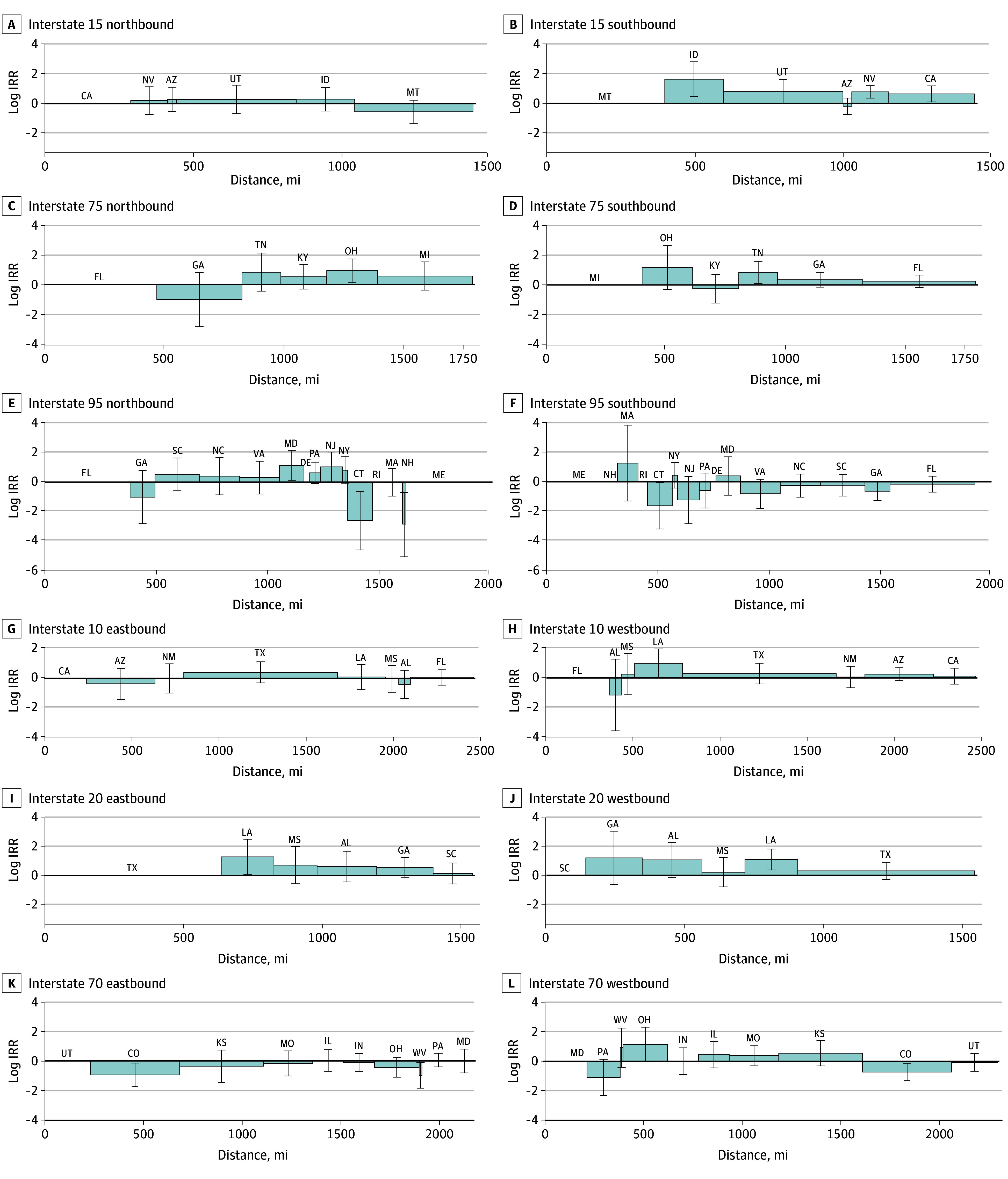
Select Transect Graphs of Associations Between Interstate Highway Connections and Traced Gun Transfers Graphs for interstate highways that cross at least 5 states and had at least 1 supported association with the count of transferred traced guns in model 3 are shown. Vertical lines represent each destination state located along the interstate of interest, organized by location along the highway in the direction specified. The y-axes represent the natural log of the incidence rate ratio (IRR) for the association of each specified directional highway variable with the count of guns recovered in the destination state. For I-95 northbound, estimates for Maine, Delaware, and Rhode Island are not reported due to unstable output from spatial models as a result of low firearm recovery counts in those states. Estimates for these states as well as New Hampshire are similarly not reported for I-95 southbound.

Figure 2 includes selected transect graphs for interstates that cover at least 5 states and had at least 1 supported association with the count of transferred traced guns in model 3. Three highways that flow northbound and southbound (I-15, I-75, and I-95) and 3 that flow eastbound and westbound (I-10, I-20, and I-70) were selected. No supported associations were found for states along I-15 northbound. Along I-15 southbound, 3 states had supported IRRs. The count of guns recovered in California from source states connected to California via I-15 southbound was greater than the count of guns recovered from states not connected to California via I-15 southbound in the fully adjusted model (IRR, 1.90; 95% CrI, 1.10-3.27). Nevada (IRR, 2.18; 95% CrI, 1.43-3.32) and Idaho (IRR, 5.15; 95% CrI, 1.59-16.75) also had supported positive associations for I-15 southbound. Ohio had a supported positive association for I-75 northbound (IRR, 2.59; 95% CrI, 1.18-5.70), while Tennessee had a positive association for I-75 southbound (IRR, 2.35; 95% CrI, 1.11-4.97). Along I-95 northbound, 2 states had supported positive associations: Maryland (IRR, 3.07; 95% CrI, 1.09-8.61) and New Jersey (IRR, 2.80; 95% CrI, 1.01-7.68). Two states along this route had negative associations: Connecticut (IRR, 0.07; 95% CrI, 0.01-0.52) and New Hampshire (IRR, 0.06; 95% CrI, 0.01-0.49). Supported negative associations were found for the I-95 southbound route for both Connecticut (IRR, 0.20; 95% CI, 0.04-0.95) and Georgia (IRR, 0.53; 95% CrI, 0.28-0.99). No supported associations were found for the recovery states along I-10 eastbound; however, Louisiana had supported positive associations for I-10 westbound (IRR, 2.70; 95% CrI, 1.02-7.16), I-20 eastbound (IRR, 3.51; 95% CrI, 1.05-11.58), and I-20 westbound (IRR, 2.93; 95% CrI, 1.44-5.94). Colorado was the only state with supported associations (both negative) for I-70 eastbound (IRR, 0.39; 95% CrI, 0.17-0.87) and I-70 westbound (IRR, 0.48; 95% CrI, 0.27-0.87).

Seven highways were not included in Figure 2: I-90, I-80, I-40, I-94, I-35, I-5, and I-25. Results for these interstates are shown in the eFigure in Supplement 1. No supported associations were found in either direction for I-90, I-80, I-40, I-94, or I-5 or for I-35 northbound or I-25 northbound. Supported positive associations were found for I-35 southbound for Kansas (IRR, 3.76; 95% CrI, 1.64-8.60) and I-25 southbound for Colorado (IRR, 4.57; 95% CrI, 2.28-9.12).

## Discussion

This study identified that between 2010 and 2019, guns flowed routinely between states along multiple transportation routes. We discovered previously unidentified Iron Pipelines, such as I-15 southbound, which had positive associations with the count of traced guns transferred into Idaho, Nevada, and California. For some states, multiple highways were involved in traced gun inflow; for example, the count of guns recovered in Louisiana was positively associated with connection to I-10 westbound, I-20 eastbound, and I-20 westbound. We also found that gun traffic along I-95, known colloquially as an Iron Pipeline, may be more complex than previously recognized. While positive associations with I-95 northbound were found for the count of traced guns transferred into Maryland and New Jersey, 2 states along this route, Connecticut and New Hampshire, had negative associations, suggesting guns may be coming into these states via other routes. These results support the hypothesis that firearms used in crimes are more likely to be transferred between states connected via major interstate highways.

This study builds on previous evidence for the flow of firearms along major highways^12,14^ by adding additional methodologic rigor. As indicated by the model findings in Table 2, inclusion of spatial and temporal factors as well as the gravity function impacted the results. By incorporating spatial methods into our analysis, our study provides an innovative examination of the ways in which highway connections may facilitate interstate transfers of guns used in crimes beyond what would be expected by spatial proximity alone. Importantly, we also found that associations sometimes differed when examining the same highway from different directions. While several states had positive associations with the count of traced gun transfers for I-15 southbound, no such associations existed for the northbound direction. Similarly, no positive associations were found for I-95 southbound, in contrast to the I-95 northbound findings. This result suggests that there may be established sources and destinations for firearms along this route that drive directionality of gun traffic. These findings align with previous literature indicating that gun flow is driven by a variety of factors, including firearm laws in each state,^9,10,11^ and suggest that there may be a synergistic relationship between interstate connection and gun laws in determining trafficking patterns.

By identifying highway routes regularly used for transfer of guns used in crimes, this study provides law enforcement and public health authorities with critical areas for intervention. While restrictive local laws have been effective in creating an additional burden for gun traffickers, necessitating interstate distribution of firearms, national policies and interstate cooperation are needed to limit gun flow and ultimately prevent thousands of firearm injuries and mortalities each year.^2^ Future research should examine interventions to reduce gun crime through the curtailment of interstate gun transfers. One option may be increased law enforcement traffic stops, which have the potential to be discriminatory,^32^ but more effective options may include supply-reduction strategies in origin states and demand-reduction strategies in destination states.

### Limitations

This study has several limitations. First, because the analysis was ecological, findings can only be interpreted at the state level and are not applicable to individuals or smaller geographic scales. Second, we only included data for the 48 contiguous states, limiting generalizability to noncontiguous US states and territories. Third, the ATF data included only recovered guns used in or suspected to have been used in crime. These data are not fully representative of all guns traveling and contributing to morbidity and mortality interstate, and this unquantified missingness of data has the potential to bias our findings. Despite this, the data offer valuable insights into the origins of interstate crime guns, a key public health intervention point. Fourth, while multiple comparisons are typically not problematic in bayesian analysis due to the more conservative nature of the bayesian CrI,^33^ there is still the possibility of drawing false conclusions with multiple testing. Fifth, while interstate highways are an important component,^21^ they do not fully capture the complex transportation network throughout the country.

## Conclusions

This study identified multiple Iron Pipelines throughout the US and showed that the use of highways in the interstate gun trafficking network may be more complex than previously recognized. Limiting gun trafficking along major interstate highways may help prevent violence, injury, and death as well as reduce long-term mental and physical consequences of exposure to violence.

## Notes

Supplement 1. **eFigure.** Selected Transect Graphs of Associations of Interstate Highway Connections With Traced Gun Transfers

Supplement 2. **eTable.** Full Set of Model 3 Results

Supplement 3.
Data Sharing Statement
