# Supplementary material for: Interstate Highway Connections and Traced Gun Transfers Between the 48 Contiguous United States
Source: JAMA Netw Open. 2024 Apr 9;7(4):e245662. doi: 10.1001/jamanetworkopen.2024.5662 (PMC11004838; doi:10.1001/jamanetworkopen.2024.5662)
Supplement: Supplement 1. — eFigure. Selected Transect Graphs of Associations of Interstate Highway Connections With Traced Gun Transfers [file jamanetwopen-e245662-s001.pdf]

Supplementary Online Content

Roberts L, Hoofnagle MH, Bushover B, et al. Interstate highway connections and traced gun transfers between the 48 contiguous United States. *JAMA Netw Open*. 2024;7(4):e245662. doi:10.1001/jamanetworkopen.2024.5662

eFigure. Selected Transect Graphs of Associations of Interstate Highway Connections With Traced Gun Transfers

This supplementary material has been provided by the authors to give readers additional information about their work.

© 2024 Roberts L et al. *JAMA Network Open*.

eFigure 1. Selected transect graphs of associations of interstate highway connections with traced gun transfers

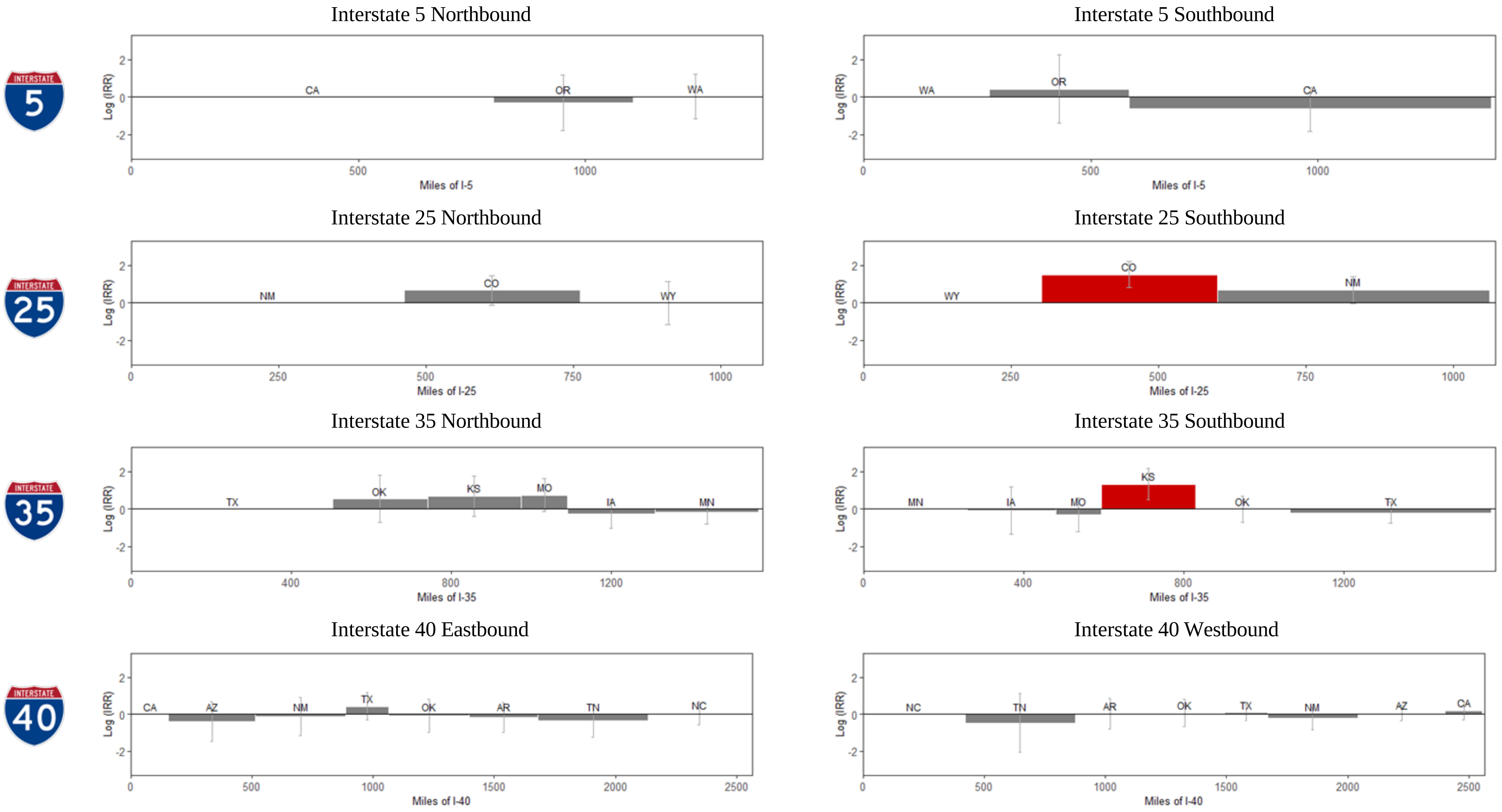

© 2024 Roberts L et al. *JAMA Network Open*.

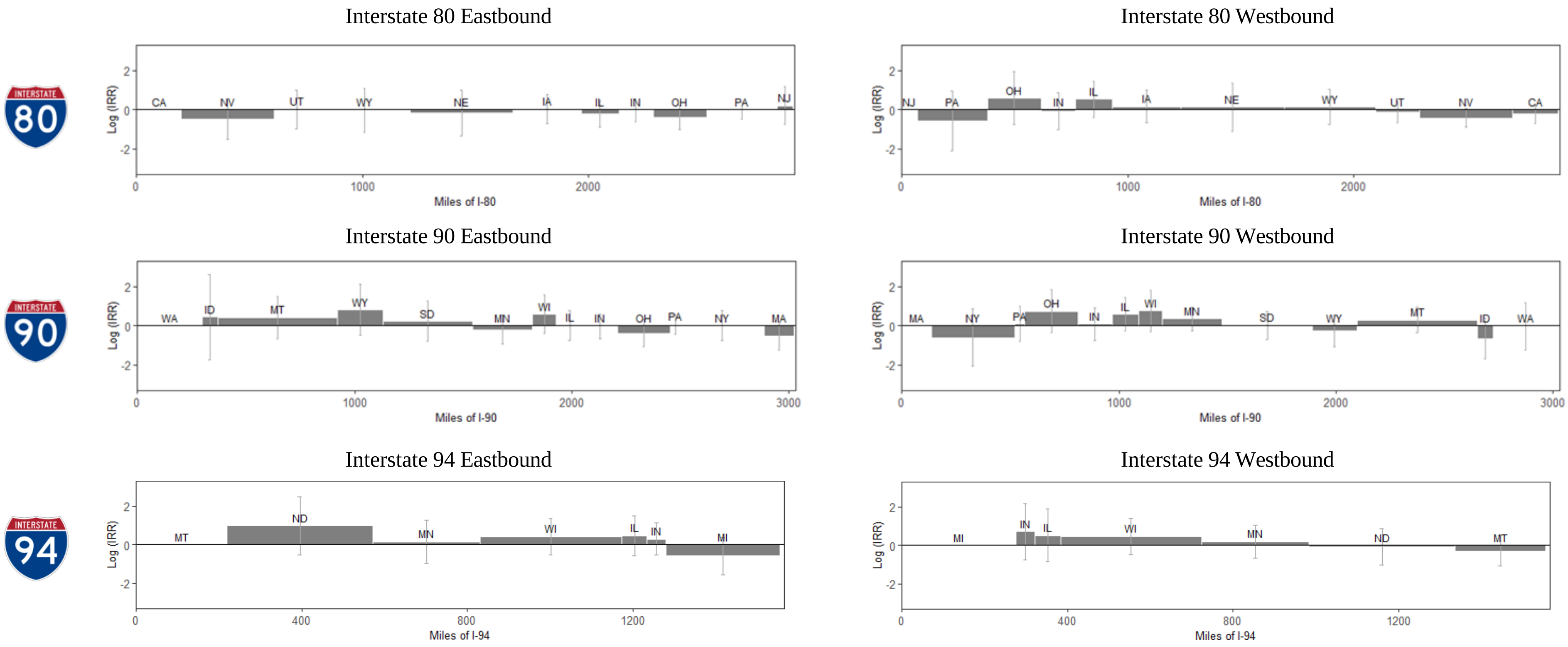

© 2024 Roberts L et al. *JAMA Network Open*.
