# Supplementary material for: Interstate Highway Connections and Traced Gun Transfers Between the 48 Contiguous United States
Source: JAMA Netw Open. 2024 Apr 9;7(4):e245662. doi: 10.1001/jamanetworkopen.2024.5662 (PMC11004838; doi:10.1001/jamanetworkopen.2024.5662)
Supplement: Supplement 3. — Data Sharing Statement [file jamanetwopen-e245662-s003.pdf]

Data Sharing Statement

Roberts. Interstate Highway Connections and Traced Gun Transfers Between the 48 Contiguous United States. *JAMA Netw Open*. Published April 09, 2024. doi:10.1001/jamanetworkopen.2024.5662

Data
Data available: Yes
Data types: Data (not involving human participants)
How to access data: cm3820@cumc.columbia.edu
When available: With publication

Supporting Documents
Document types: None

Additional Information
Who can access the data: Anyone requesting the data
Types of analyses: For any purpose
Mechanisms of data availability: Upon request
